# Expectation-propagation for weak radionuclide identification at radiation portal monitors

**DOI:** 10.1038/s41598-020-62947-3

**Published:** 2020-04-22

**Authors:** Yoann Altmann, Angela Di Fulvio, Marc G. Paff, Shaun D. Clarke, Mike E. Davies, Stephen McLaughlin, Alfred O. Hero, Sara A. Pozzi

**Affiliations:** 1 School of Engineering and Physical Sciences, Heriot-Watt University, Riccarton, Edinburgh, EH14 4AS United Kingdom; 2 Department of Nuclear, Plasma, and Radiological Engineering, University of Illinois at Urbana-Champaign, Urbana, IL 61801 United States; 3 Los Alamos National Laboratory, Los Alamos, NM 87545 United States; 4 Department of Nuclear Engineering and Radiological Sciences, University of Michigan, Ann Arbor, MI 48109 United States; 5 School of Engineering University of Edinburgh King’s Buildings, Edinburgh, EH9 3JG United Kingdom; 6 Department of Electrical Engineering and Computer Science, University of Michigan, Ann Arbor, MI 48109 United States

**Keywords:** Engineering, Mathematics and computing, Physics

## Abstract

We propose a sparsity-promoting Bayesian algorithm capable of identifying radionuclide signatures from weak sources in the presence of a high radiation background. The proposed method is relevant to radiation identification for security applications. In such scenarios, the background typically consists of terrestrial, cosmic, and cosmogenic radiation that may cause false positive responses. We evaluate the new Bayesian approach using gamma-ray data and are able to identify weapons-grade plutonium, masked by naturally-occurring radioactive material (NORM), in a measurement time of a few seconds. We demonstrate this identification capability using organic scintillators (stilbene crystals and EJ-309 liquid scintillators), which do not provide direct, high-resolution, source spectroscopic information. Compared to the EJ-309 detector, the stilbene-based detector exhibits a lower identification error, on average, owing to its better energy resolution. Organic scintillators are used within radiation portal monitors to detect gamma rays emitted from conveyances crossing ports of entry. The described method is therefore applicable to radiation portal monitors deployed in the field and could improve their threat discrimination capability by minimizing “nuisance” alarms produced either by NORM-bearing materials found in shipped cargoes, such as ceramics and fertilizers, or radionuclides in recently treated nuclear medicine patients.

## Introduction

The growing terrorism threat based on the use of special nuclear materials (SNMs), i.e., highly enriched uranium (HEU), weapons-grade plutonium (WGPu), or high-activity radiological sources has reinforced the need for improved population protection mechanisms. Nuclear security aims to deter and detect the smuggling of these materials across state borders. One major defense mechanism involves the installation of radiation portal monitors (RPMs) at border crossings. These RPMs typically consist of ^3^He proportional counters embedded in polyethylene for neutron detection, and slabs of polyvinyl-toluene (PVT) scintillators for gamma-ray detection. Only a tiny fraction of the millions of vehicles and cargo containers entering a country like the United States are likely to be carrying radiological contraband. The International Atomic Energy Agency’s Incident and Trafficking Database (ITDB) merely counts a few dozen reported successful interdictions of nuclear and radiological materials globally per year^1,2^. The ITDB provides only a partial picture of the number of smuggling attempts. The reported figures should be considered a lower bound of the number of successful interdictions, because they include only successful interdictions, voluntarily reported by the member states.

Complicating matters, the radiological contraband might be well shielded. In 2017, the United Nations Conference on Trade and Development estimated the global container port throughput at over 750 million 20-foot equivalent units^3^. As a consequence, RPMs are limited in measurement time to minimize unnecessary impediments to the flow of traffic and commerce. RPMs need to function rapidly while collecting sufficient data to positively identify the presence of a radiation source, which may produce a signal just slightly above the natural background.

Border protection agents screen inbound vehicles and cargo containers for suspicious levels of radiation relative to the background, and flag these for a more thorough secondary inspection. Detecting smuggled nuclear and radiological material is analogous to finding a needle in a haystack, whereby SNMs can be difficult to detect, quantify, and locate. Nuisance alarms are radiation alarms caused by sources of radiation that pose no security threat. Many common goods shipped across border crossings contain sufficient naturally occurring radioactive material (NORM) to set off gamma alarms in RPMs^4^. Medical isotopes are another growing source of nuisance alarms. A patient may emit sufficient gamma radiation for days or even weeks after a procedure to set off an RPM gamma alarm^4–7^, depending on the nuclear medicine isotope used and its administered activity.

NORM-bearing cargo and nuclear medicine patients are significantly more prevalent than nuclear smugglers in cross-border traffic. Hence, customs and border protection agents spend an exorbitant amount of time processing nuisance alarms in secondary inspections that can last tens of minutes per offending vehicle or cargo container^8^. Due to the low signal to background ratio, simply alarming on the presence of a radioactive source is a challenge in itself for primary inspection. In the interest of saving time for both customs and border protection agents, as well as people crossing borders, combining primary and secondary inspections appears as an attractive solution. In an ideal scenario, the primary inspection would simultaneously detect, identify and quantify any source of radiation of interest, so that, for example, nuclear medicine patients avoid the discomfort caused by a lengthy secondary inspection. Identifying radionuclides, however, is even more sensitive to signal-to-background ratio than simply detecting the presence of a radiation source.

One concerning and challenging scenario involves the contextual presence of multiple radionuclides, i.e., mixed sources. In this case, strong NORM sources can mask a weaker SNM source, and further jeopardize the identification process. Gamma-ray spectroscopy inspections performed using inorganic scintillators or semiconductor detectors, such as NaI(Tl) or HPGe, respectively, are typically able to resolve most of the photopeaks, which serve as fingerprints of the present radionuclides, and therefore facilitate the nuclide identification in a mixed source scenario^9^. The vast majority of deployed RPMs utilizes instead organic scintillators, i.e., PVT, because of the high intrinsic efficiency of these detectors, their relatively low cost, and suitability to be produced in large shapes. The response of organic scintillator-based RPMs is not characterized by sharp photopeaks, but rather by smooth edges and continuum regions that result from Compton scattering interactions. Therefore, the spectral response of an RPM organic scintillator to a mixed source will essentially be a smooth linear combination of the responses to individual sources. It is hence challenging to identify all the components of the mixed source and estimate the relative activities of the constituent sources.

The performance of a portal monitor in terms of sensitivity, i.e., maximization of the positive detection rate, is a function of the detection efficiency of the system and its form factor, which should be optimized for a specific application. Paff and colleagues^8^ have already shown that the system sensitivity can be optimized by selecting large detector panels. In this work, we focus on the capability of identifying multiple sources in a mixture of nuclides, following an alarm event.

The proposed method is also relevant to a number of other radiation identification and localization applications, such as radionuclide search with unmanned vehicles in a given environment, where the statistics of the signal of interest is poor compared to the background, because of short measurement time, distance between the detector and the source, low detection intrinsic efficiency and/or weakness of the source.

## Algorithms for RPM signal unmixing

Radiation detection and characterization in the nuclear security area is challenging due to the low intensity of the signal of interest, typically much lower than the background. Two main detrimental components are added to the SNM signal of interest: spectra of additional NORM sources, either located inside the cargo or part of the natural background surrounding the portal monitor, and intrinsic observation Poisson noise (shot noise), which is not negligible for short measurement times and therefore can lead to poor signal-to-noise ratios. Bayesian inference is particularly attractive in such challenging scenarios, and advances in approximate methods^10,11^ allow complex models to be used with computational times compatible with real-time constraints.

Bayesian approaches to detect, classify, and estimate smuggled nuclear and radiological materials are not a new consideration^6,12^, and were extensively studied for the development of the Statistical Radiation Detection System at Lawrence Livermore National Laboratory. This group has used Bayesian model-based sequential statistical processing techniques to overcome the low signal-to-background ratio that complicates traditional gamma spectroscopy techniques with high-resolution HPGe and inorganic scintillation detectors^13,14^. Bayesian approaches have also been applied to radionuclide identification for NaI(Tl) detectors using a wavelet-based peak identification algorithm with Bayesian classifiers^15^, for LaBr_3_(Ce) using a sequential approach^16^, and to HPGe detectors using non-parametric Bayesian deconvolution to resolve overlapping peaks^17^. Bayesian approaches have been recently investigated for the detection of single and mixed gamma sources with short measurement times^12^. The use of related machine-learning-based methods was also recently demonstrated for source identification in spectra recorded using inorganic scintillators^18^.

## Results

**Figure 1:**
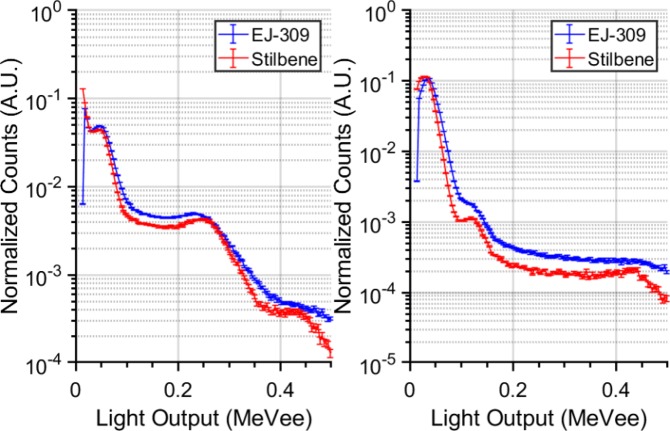
Comparison of light output spectra of ^201^Tl (left) and ^99*m*^*Tc* (right) sources measured using the EJ-309 (blue curves) and the stilbene (red curves) scintillators. For comparison purposes, the spectra have been normalised to integrate to one.

In this study, we considered two types of organic scintillation detectors, based on liquid EJ-309 and stilbene crystal, respectively, as detailed in the “Methods” section. The functional difference between the two detectors most relevant to this work is their energy resolution as illustrated in Fig. 1, which depicts their integral-normalized response to a ^201^Tl (left) and to a ^99*m*^Tc (right) source. This figure shows that stilbene exhibits sharper Compton edges than EJ-309, thanks to its better energy resolution.

Table 1 lists the 11 nuclides that were measured using the two different detectors, and the relative fractions used to generate synthetic mixtures and assess the performance of the new algorithm. For each mixture, several data sets were created to obtain spectra with a total counts from $500$ to $500$ k, where the observation noise was modeled by Poisson noise.

**Table 1:** Composition of the nine mixtures tested in this work.

	^241^Am	^133^Ba	^57^Co	^137^Cs	^123^I	^201^*Tl*	^67^Ga	I^131^	^99*m*^Tc	^111^In	WGPu
Mixture 1	1/6	0	0	5/6	0	0	0	0	0	0	0
Mixture 2	0	1/3	0	1/3	0	0	1/3	0	0	0	0
Mixture 3	0	0	0	0	0	0	1/3	0	1/3	0	1/3
Mixture 4	0	1/4	0	1/4	1/4	0	0	0	1/4	0	0
Mixture 5	0	1/2	0	0	0	0	0	1/3	0	0	1/6
Mixture 6	0	0	0	0	0	0	0	0	5/6	0	1/6
Mixture 7	0	0	0	0	0	0	0	0	6/7	0	1/7
Mixture 8	0	0	0	0	0	0	0	0	7/8	0	1/8
Mixture 9	0	0	0	0	0	0	0	0	8/9	0	1/9

The fractions represent the ratio of the detected counts associated with each source.

We compared the unmixing performance of the new algorithm, referred to as MMSE_*BTG*_, to that of two Bayesian strategies, namely the maximum a-posteriori (MAP) and the minimum mean squared error (MMSE_*L*1_) approaches presented in^12^. These two approaches, denoted by MAP_*L*1_ and MMSE_*L*1_, respectively, are detailed in the “Methods” section.

**Figure 2:**
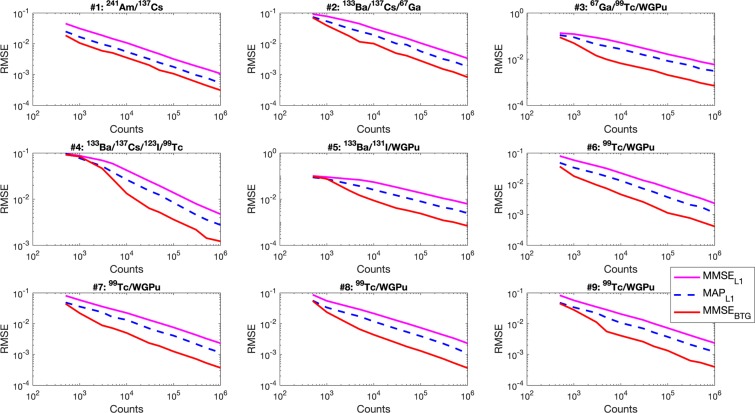
RMSEs obtained with the MMSE_*BTG*_, MMSE_*L*1_ and MAP_*L*1_ algorithms for the mixtures described in Table 1 and measured with the stilbene detector, as a function of the detection counts.

As the metric for estimation accuracy, we used the root-mean-square error (RMSE)

1 $$RMSE=\sqrt{\frac{\parallel {\bf{z}}-\hat{{\bf{z}}}{\parallel }_{2}^{2}}{N}}$$

between the known nuclide fractions **z** and their estimated values $\hat{{\bf{z}}}$, where *N* is the number of nuclides in the spectral library. Figure 2 compares the RMSEs obtained by the three methods mentioned above for the nine mixtures of Table 1, as the total number of counts increases (from $500$ to $1$ M) and using the stilbene detector. The new MMSE_*BTG*_ method generally provides more robust results, compared to the MAP_*L*1_ and MMSE_*L*1_ approaches, yielding consistently lower RMSEs. The MMSE_*BTG*_ RMSE becomes comparable to the MAP_*L*1_ RMSE when only 500 counts are measured, and when the mixture contains nuclides with spectral similarities, e.g., ^123^I and ^99*m*^Tc in the fourth mixture.

**Figure 3:**
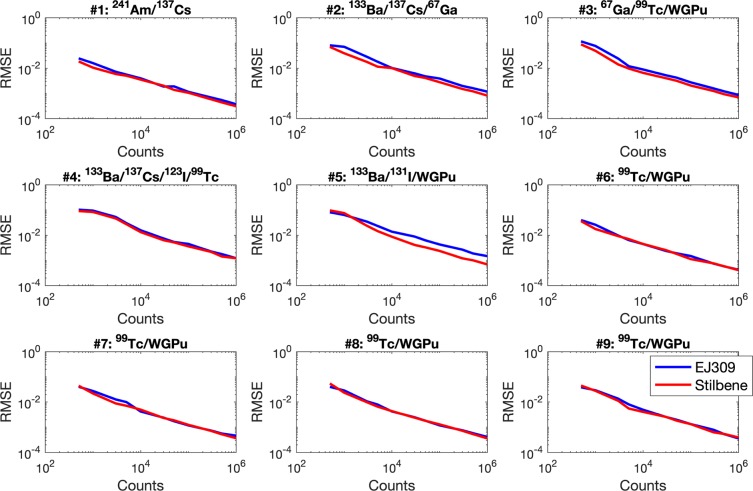
RMSE of the MMSE_*BTG*_ algorithm calculated using the stilbene (red curves) and EJ-309 (blue curves) experimentally measured data.

The RMSEs obtained using the MMSE_*BTG*_ algorithm and simulated data show overall comparable performances using either detector (see Fig. 3). The results using the stilbene detector are slightly better, i.e., present lower RMSEs, especially for mixtures of three or more nuclides, e.g., WGPu, ^99*m*^Tc, and ^67^Ga (mixture 3). This result is expected because of the better energy resolution of stilbene, compared to EJ-309. Similar results have been obtained with the two other competing methods.

**Figure 4:**
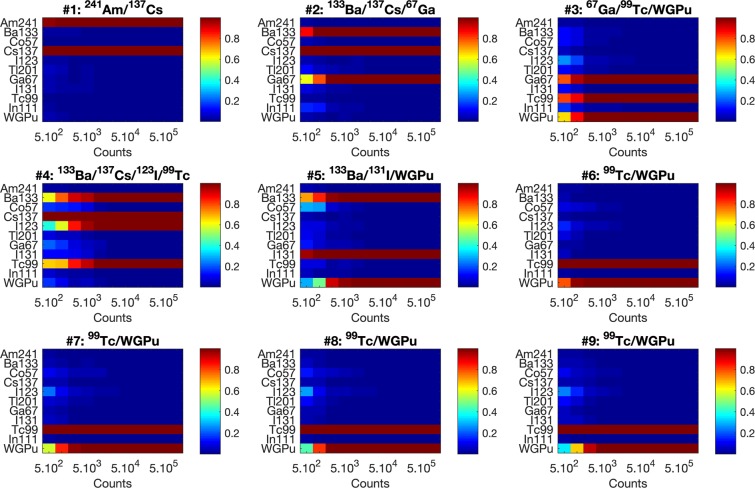
Estimated marginal posterior probabilities of the presence of each of the library sources in the mixture as a function of the total photon count. The probabilities depicted have been obtained using the stilbene detection spectra, have been computed individually across $100$ noise realizations, and finally averaged over these $100$ noise realizations.

A significant advantage of the proposed MMSE_*BTG*_ algorithm is that it directly provides uncertainty quantification, i.e., the estimated probability of the presence of each source from the library. The MMSE_*BTG*_ algorithm generates theses estimates from the posterior distribution, which are not directly available from the MAP_*L*1_ and MMSE_*L*1_ algorithms. If the measured spectrum consists of more than 1000 counts, the algorithm correctly identifies with high probability the nuclides in the mixture and its performance slightly degrades as the number of sources that are present increases and the overall gamma counts per source decrease (see Fig. 4). In addition to providing estimated probabilities of source presence, the MMSE_*BTG*_ yields superior performance, compared to the MAP_*L*1_ and MMSE_*L*1_ algorithms used in Fig. 2. Furthermore, in contrast to the proposed MMSE_*BTG*_ approach, the MMSE_*L*1_ and MAP_*L*1_ algorithms require tuning of a threshold for source detection, whose optimal value (in terms of probabilities of false alarm and detection) is difficult to tune in practice, as it depends on the counts and the mixture composition. For this reason, we only report here the detection results obtained using the MMSE_*BTG*_ approach.

**Figure 5:**
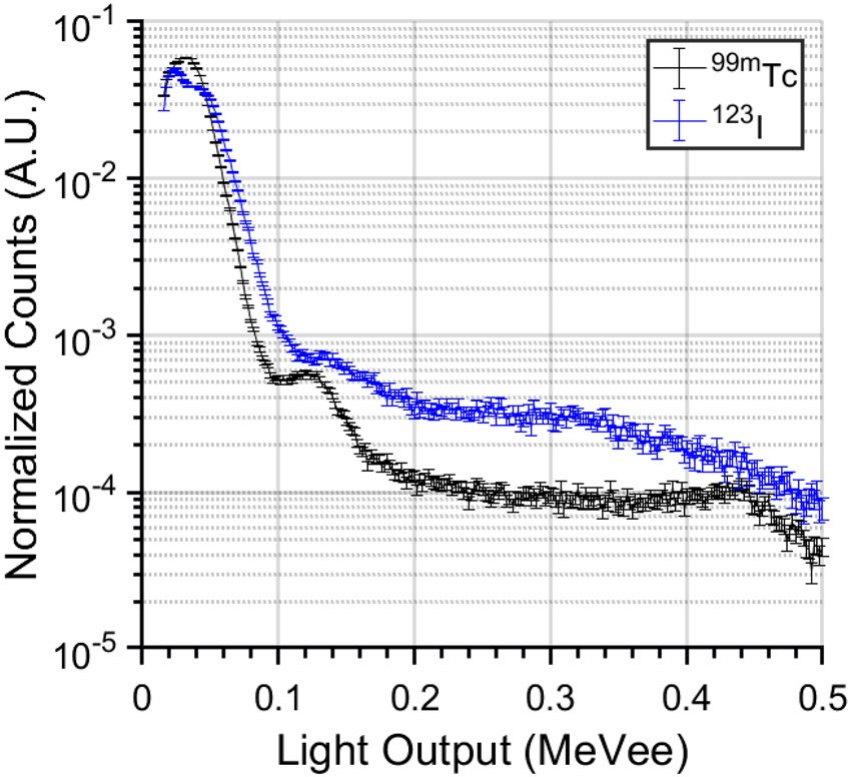
Comparison of integral-normalized light output spectra emitted by ^123^I and ^99*m*^*Tc* measured by the EJ-309 detector. The zero-lagged cross-correlation coefficient between the two spectra is 0.97.

In Fig. 4, an increasing number of isotopes not present in the actual mixture is identified as potentially present, when there are few gamma counts. For example, for sparse spectra (<1,000 counts) containing WGPu and ^99*m*^Tc (mixtures 6–9), the algorithm suggests the potential presence of ^123^I. This can be explained by the similarity of the spectra of ^123^I and ^99*m*^Tc (as shown in Fig. 5). The discrimination of these two nuclides becomes easier as the gamma counts increase.

Mixtures 6–9 simulate a specific scenario, where a WGPu source is detected together with an increasing amount of ^99*m*^Tc, which is the most commonly used medical radioisotope and could, therefore, be used to mask (in terms of relative counts) the presence of WGPu. The results illustrate that the estimated probability of presence of WGPu decreases as its proportion decreases in the mixture (from mixture 6 to mixture 9), as could be expected.

**Figure 6:**
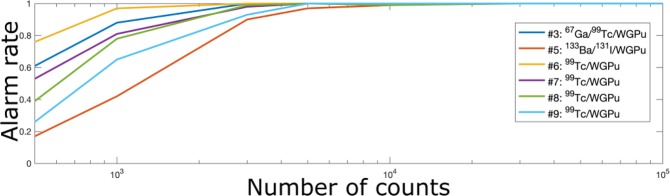
Comparison of the WPGu alarm rates with the stilbene detector in the presence of mixtures with WGPu.

Figure 6 shows the empirical WGPu alarm rate, i.e., the fraction of the measurements containing WGPu for which the estimated probability of WGPu presence is larger than 50%, as a function of the total photon counts (top) and WGPu counts (bottom) for the different WGPu -based mixtures, using the stilbene detector. With a target WGPu alarm rate of 80%, a few hundreds of counts from the WGPu source would set off the portal alarm, even in the presence of up to three other highly-radioactive masking sources. The highest number of approximately 3000 overall counts to trigger an alarm state is needed for mixture 5, which includes WPGu, ^133^Ba, and ^131^In. In similar irradiation conditions, in the presence of a mixed source, a detector similar to the one investigated would record approximately 130 counts during a 3-s vehicle scan time^8^. Assuming that the intrinsic efficiency scales with the volume of the detector and factoring an efficiency loss of 10$\%$ due to non-ideal light collection, a relatively small 2752 *cm*^3^ single module used in portal monitors^19^ would record approximately 3100 counts during a 3-s acquisition of a mixture of ^133^Ba, ^131^In, and WGPu. This acquisition time would be sufficient to set an alarm condition in the portal monitor. Regarding computational costs, the three competing methods (MMSE_*BTG*_, MMSE_*L*1_ and MAP_*L*1_) have been implemented using Matlab 2017b running on a MacBook Pro with 16 GB of RAM and a 2.9 GHz Intel Core i7 processor. Since the MMSE_*L*1_ is a simulation-based algorithm (see “Methods” section), its computational cost is significantly higher than the two other methods and it requires 66 s to analyze one spectrum (using 5000 iterations and assuming at most 11 sources in the mixture), on average. This prevents its use within portal monitors. Conversely, MAP_*L*1_ only takes 50–110 ms per spectrum and is the fastest method. Our new algorithm MMSE_*BTG*_ is slower (approximately 1 s per spectrum) but still compatible with real-time monitoring. While slower than MAP_*L*1_, MMSE_*BTG*_ provides better estimates and allows automatic source detection and uncertainty quantification.

## Discussion

RPMs must be able to detect weak SNM sources masked by a stronger NORM or nuisance radiation source. In this work, we overcame the limited energy resolution of organic scintillators by applying a new Bayesian algorithm to decompose and identify mixed gamma-ray sources. Bayesian algorithms proved to be useful tools to improve the source detection accuracy even with limited statistics (few counts) and poor signal-to-background ratios.

The proposed Bayesian MMSE_*BTG*_ technique is designed to allow more accurate source identification and quantification in the presence of one or more masking nuclides, with cumulative count integrals as low as 500 counts. The automated identification obtained with the MMSE_*BTG*_ method is more robust than using the MAP_*L*1_ and MMSE_*L*1_ algorithms, which require unpractical parameter tuning. The main benefit of the proposed method is a more sensitive model that captures the sparsity of the mixing coefficients. The application of the MMSE_*BTG*_ reduces, for instance, the average root-mean-square error between real and estimated nuclide fractions to $0.0177$, compared to $0.0334$ for MAP_*L*1_, and $0.0584$ for MMSE_*L*1_ for the sixth mixture, containing ^99*m*^*Tc* and WGPu, with only $1000$ detection events. Our study also confirmed the importance of detector energy resolution. The stilbene crystal exhibits a better energy resolution than EJ-309 and, as a result, the stilbene data yielded a slightly better quantification accuracy, compared to EJ-309. Therefore, a slight improvement in the nuclide identification accuracy can be achieved by improving the energy resolution of the detector. Energy resolution improvement can be achieved either by using different materials, as we have shown in this study, and also by optimizing the detector’s light collection geometry^20^. A relevant feature of organic scintillators is their sensitivity to both neutrons and gamma rays. Neutron and gamma-ray interactions in the organic scintillators are distinguishable through pulse shape discrimination. The neutron signature was not used in this work but could be further exploited to aid the classification of fissile and other neutron emitting materials.

In this paper, we have applied new Bayesian algorithms for the identification of source mixtures that are not shielded. While this scenario applies to pedestrian portal monitors, it would be interesting to study the algorithm performance when sources are transported with other goods, or deliberately shielded. Effectively shielding SNMs and intense gamma-ray emitting radionuclides, such as ^137^*Cs* and ^60^*Co*, would require a combination of low- and high-atomic-number elements. The current algorithm could be enhanced by coupling it to spectra reconstruction methods that we have recently developed^21^, to account for the spectral effect of shielding materials, given their known gamma-ray and neutron attenuation coefficients, as proposed by Lawrence and colleagues^22^. It should also be noted that containers carrying covert or overt amounts of metal are likely to prompt secondary inspections. For example, cargo containers carrying a large number of metal items typically undergo radiation inspection because orphan sources are often improperly disposed of as scrap metal and can be cast into metal parts^23^. Conversely, electro-magnetic inspection is performed on cargoes that are declared metal-free, and would promptly identify covert metal items.

## Methods

Over the past years, our group has developed several radionuclide identification algorithms for EJ-309-based portal monitors^6,8,12^. This work proposes a novel computational Bayesian method for source identification that we have applied to both liquid EJ-309 and solid-state trans-stilbene scintillators. In this section, we first detail how our measured data have been collected and then the principle of the new computational method.

### Experimental methods

We have used two detectors: an EJ-309 organic liquid scintillator (7.6-cm diameter by 7.6-cm height) by Eljen Technology, and a cylindrical trans-stilbene crystal (5.08-cm diameter by 5.08-cm height) produced using the solution-growth technique by Inrad Optics. The detection system used can be easily scaled up to be a pedestrian portal by using an array of detector cells. Despite the similar composition, EJ-309 and stilbene exhibit different properties (see Table 2). Noticeably, EJ-309 has a higher scintillation efficiency and higher density, compared to stilbene, which determines its higher intrinsic detection efficiency^24^. However, the stilbene crystal shows a favorable energy resolution, defined as the full width at half maximum (FWHM) of a spectrum peak in response to the energy deposited in the detector by monoenergetic charged recoils, divided by its centroid. This improved energy resolution can enhance isotope identification accuracy using stilbene over EJ-309. Note that the energy resolution of a scintillation detector is affected by both the scintillating material and the light collection and conversion process. The energy resolution at 478 keVee of stilbene and EJ-309 detectors of the same size as those used in this work is 9.64 ± 0.06^25^ and 19.33 ± 0.18^26^, respectively.

**Table 2:** Physical properties of EJ-309 and stilbene detectors.

	EJ-309^39^	Stilbene^40,41^
Chemical Formula	n.a.	*C*_14_*H*_12_
Geometric isomerism	n.a.	Trans
H:C ratio	1.25	0.86
Density (g *cm*^−3^)	0.959	1.15
Light output (% anthracene)	80%	120%
Maximum wavelength (nm)	424	382
Scintillation efficiency (photons/1 MeVee)	12300	9760
Flash point (°*C*)	144	n.a.

**Figure 7:**
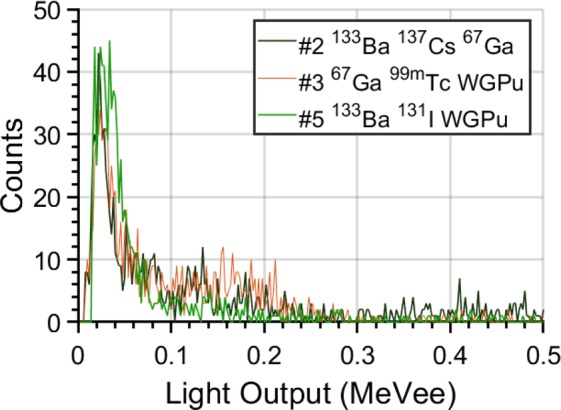
Comparison of light output spectra of mixtures 2, 3, and 5. The total number of counts in each distribution is approximately 1000.

For completeness, Fig. 7 depicts the light output spectra of some of the mixtures analyzed, when approximately 1000 counts were acquired. Despite the spectra consisting of different nuclides, their overall distribution as a function of light output is similar. This effect is due to the scatter-based detection of organic scintillators and the low counting statistics.

We measured a variety of sources, including ^241^*Am*, ^133^*Ba*, ^57^*Co* and ^137^*Cs* sources with activities of approximately 500 kBq. The WGPu source (180 MBq) was measured at the Zero-Power Research Reactor of the Idaho National Laboratory^27^. In addition, 260 kBq liquid solution samples of the medical isotopes, i.e., ^99*m*^*Tc*, ^111^*In*, ^67^*Ga*, ^123^*I*, ^131^*I*, and ^201^*Tl* were measured at the University of Michigan C.S. Mott Children’s Hospital.

The on-the-fly radionuclide identification algorithms used in this work rely on a library of nuclides that is assumed to include the species potentially present in the mixtures. The detection of unknown sources is out of the scope of this work and is left for future work. The isotope library used in this work consists of a collection of light output spectra acquired over one hour to reduce shot-noise effects. As the two detectors exhibit slightly different light responses, calibration was necessary to detect the same portion of the energy spectrum with both detectors. The detectors were gain-matched using a 3.3-MBq ^137^*Cs* source, by aligning the ^137^*Cs* Compton edge to 1.8 V in the pulse-height detector response. Lower and upper detection thresholds of 40 keV-electron-equivalent (keVee) and 480 keVee, respectively, were applied to both stilbene and EJ-309 detectors light output spectra. The electron-equivalent light output of a pulse in a scintillator, measured in electron-equivalent electron Volts, or eVee, refers to the energy required for an electron to produce a pulse with equivalent light output.

### Computational Method

#### Bayesian estimation: competing methods

Bayesian methods rely on exploiting the posterior distribution of variables of interest, by combining the observed data with additional prior information available about those variables. Here, we are interested in finding the coefficients associated with a set of nuclides. Numerous strategies have been proposed to solve this problem and, before introducing the proposed method, we first discuss the two methods used in^12^, namely, the MAP_*L*1_ and the MMSE_*L*1_ methods, to motivate the new MMSE_*BTG*_ method.

Consider an observed spectral response ${\bf{y}}={[{y}_{1},\ldots ,{y}_{M}]}^{T}$ observed in *M* non-overlapping energy bins ($M=232$ for all the results presented here), which is associated with a mixture of up to $N$ known sources whose individual spectral responses are denoted by ${\{{{\bf{A}}}_{:,n}\}}_{n=1,\ldots ,N}$ and gathered in the $M\times N$ matrix ${\bf{A}}=[{{\bf{A}}}_{:,1},\ldots ,{{\bf{A}}}_{:,N}]={[{{\bf{A}}}_{1,:}^{T},\ldots ,{{\bf{A}}}_{M,:}^{T}]}^{T}$. Each **A**_*m*,:_ is a row vector gathering the spectral responses of the $N$ known sources in the *m*th energy bin. Note that the spectral signatures are normalised such that they integrate to one and that this normalization has been performed using spectra measured with long integration times to reduce as much as possible shot-noise effects during the normalization. The amount/coefficient associated with the $n$th source is denoted by *x*_*n*_ and the $N$ coefficients are gathered in the vector ${\bf{x}}={[{x}_{1},\ldots ,{x}_{N}]}^{T}$. A classical approach to source separation is to assume as a first approximation, a linear mixing model which can be expressed in matrix/vector form as ${\bf{y}}\approx {\bf{A}}{\bf{x}}$. This model assumes that all the radiation sources present in the scene that are not included in the matrix **A** can be neglected. To avoid environment-dependent results, the background is neglected here. Our aim here is to study the nuclide identification and quantification in scenarios where the integration time is short and thus when the number of gamma detection events is low. In such cases, the observation noise corrupting each measurement can be accurately modeled by Poisson noise, leading to Poissonian form of the likelihood.

2 $$f({y}_{m}|{\bf{x}})={({{\bf{A}}}_{m,:}{\bf{x}})}^{{y}_{m}}\,\text{exp}[-{{\bf{A}}}_{m,:}{\bf{x}}]/{y}_{m}!,\,\forall m=1,\ldots ,M.$$

Since **A** is known, it is omitted in all the conditional distributions hereafter. Note that Eq. (2) implies that the sources present a fixed activity (or are static) during the integration time. In more complex scenarios, more complex models such as compound Poisson models might be used. Conditioned on the value of **x**, the entries of **y** are independently distributed, i.e., $f({\bf{y}}|{\bf{x}})={\prod }_{m=1}^{M}\,f({y}_{m}|{\bf{x}})={\prod }_{m=1}^{M}\,f({y}_{m}|{{\bf{A}}}_{m,:}{\bf{x}})$. Bayesian methods for spectral unmixing rely on additional prior information available about **x** to enhance its recovery from **y**. Such methods formulate a priori information through a prior distribution $f({\bf{x}})$ and the estimation of **x** can then be achieved using the posterior distribution $f({\bf{x}}|{\bf{y}})=f({\bf{y}}|{\bf{x}})f({\bf{x}})/f({\bf{y}})$. The maximum a posteriori (MAP) estimate can be obtained by solving the following optimization problem

3 $$\hat{{\bf{x}}}=\mathop{\text{argmax}}\limits_{{\bf{x}}}\,f({\bf{x}}|{\bf{y}})$$

while the minimum mean squared error (MMSE) estimate, or posterior mean, can be obtained by computing the expectation ${E}_{f({\bf{x}}|{\bf{y}})}[{\bf{x}}]$. Using a product of independent exponential prior distributions for **x**, leads to a model that is based on an ${\ell }_{1}$-norm penalty. This is the model used in our preliminary work^12^. In that work, we compared two approaches, namely, MAP estimation and MMSE estimation, leading to two algorithms, MAP_*L*1_ and MMSE_*L*1_, respectively. It is important to mention that this choice of sparsity model is primarily motivated by the fact that the problem in (3) is convex and can be solved efficiently. While the MMSE_*L*1_ algorithm is based on Markov chain Monte Carlo (MCMC) methods and allows the estimation of a posteriori confidence intervals (which are not directly available with the MAP_*L*1_ method), we showed^12^ that the proportions estimated were generally worse than when using MAP_*L*1_. This is primarily due to the fact that although exponential prior distributions promote sparse MAP estimates; this family of distributions is not sparsity promoting (it only tends to concentrate the mass of the distribution around the origin). Hence, the resulting probabilistic estimates, such as means or covariances are questionable^28^. This observation is also confirmed with the results in Fig. 2. Our previous study^12^ also showed that by constraining $K\le N$ the maximum number of sources present in each mixture, it is possible to further improve the unmixing performance using MAP_*L*1_. This improvement however comes at a high computational cost as it requires comparing all the possible partitions of $K$ sources, out of $N$ sources in the original spectral library. This becomes rapidly intractable as $N$ increases. It also requires a level of supervision (to set $K$ properly) which is incompatible with practical, real-time applications.

#### Alternative prior model for sparse mixtures

In this work, we first propose to use an alternative, more efficient, sparsity-promoting prior model for **x**. Precisely, we consider the following Bernoulli-truncated Gaussian (BTG) model

4 $$\begin{array}{ll}f({x}_{n}|{w}_{n})=(1-{w}_{n})\delta ({x}_{n})+{w}_{n}{{\mathscr{N}}}_{{{\mathbb{R}}}^{+}}({x}_{n};0,{\sigma }_{n}^{2}), & \forall n=1,\ldots ,N\\ {f}_{n}({w}_{n}=1)={\pi }_{n}, & \forall n=1,\ldots ,N,\end{array}$$

where *δ*(·) denotes the Dirac delta function which is equal to 1 when ${x}_{n}=0$ and 0 elsewhere and where ${{\mathscr{N}}}_{{{\mathbb{R}}}^{+}}({x}_{n};0,{\sigma }^{2})$ is a truncated Gaussian distribution, defined on ${{\mathbb{R}}}^{+}$ to enforce the non-negativity of the elements of **x**. Moreover, 0 and *σ*^2^ are respectively the mean and variance of the Gaussian prior truncation. In Eq. (4), ${w}_{n}$ is a binary variable which relates to the presence (${w}_{n}=1$) or absence (${w}_{n}=0$) of the $n$th source and the probability *π*_*n*_ is the prior probability of presence of the $n$th source. More precisely, the first line in Eq. (4) reduces to a mass at 0 enforcing ${x}_{n}=0$ if ${w}_{n}=0$ (source absent) and to a truncated Gaussian distribution if ${w}_{n}=1$ (source present).

The joint prior model can then be expressed as $f({\bf{x}},{\bf{w}}{\boldsymbol{)}}={\prod }_{n=1}^{N}\,f({x}_{n}|{w}_{n}){f}_{n}({w}_{n})$ and the proposed unmixing algorithm aims at estimating jointly $({\bf{x}},{\bf{w}}={[{w}_{1},\ldots ,{w}_{N}]}^{T})$, i.e., at performing jointly the source identification (through **w**) and quantification (through **x**). Note that {*π*_*n*_}_*n*_ and $\{{\sigma }_{n}^{2}\}$ are assumed to be known here and can be used-defined. For the prior probabilities of presence, we set ${\pi }_{n}=1/N,\forall n$ as we expect a limit number of sources to be simultaneously present in the mixture, while we do not wish to promote any specific source. While arbitrary large values could in principle be used for the variances $\{{\sigma }_{n}^{2}\}$, reflecting the lack of information about the activity of the sources to be detected, this strategy can lead to poor detection^29^. If the variances cannot be set from prior knowledge, an alternative approach, adopted here consists of adjusting it using the current observation, in an empirical Bayes fashion. Since the matrix **A** is normalised, the variances $\{{\sigma }_{n}^{2}\}$ should scale with the photon counts, provided that few sources are expected simultaneously in the mixture. In this work we set ${\sigma }_{n}^{2}=0.1\,{\sum }_{m=1}^{M}\,{y}_{m}$ for each source and for all the results presented and did not observed unexpectedly poor detection results.

Using the Bayes’ rule, the joint posterior distribution of $({\bf{x}},{\bf{w}})$ is given by $f({\bf{x}},{\bf{w}}|{\bf{y}})=f({\bf{y}}|{\bf{x}})f({\bf{x}},{\bf{w}})/f({\bf{y}})$. Unfortunately, the posterior means ${E}_{f({\bf{x}},{\bf{w}}|{\bf{y}})}[{\bf{x}}]$ and ${E}_{f({\bf{x}},{\bf{w}}|{\bf{y}})}[{\bf{w}}]$ associated with this posterior distribution are intractable analytically and the traditional approach to exploit the posterior distribution consists of using a simulation method (as used in the MMSE_*L*1_ algorithm). In particular, constrained Hamiltonian Monte Carlo methods^30^ have been investigated to solve regression problems in the presence of Poisson noise^12,31^ (see also^32^ for comparison of samplers). However, efficient sampling from $f({\bf{x}},{\bf{w}}|{\bf{y}})$ is very difficult due to the Poisson likelihood (2) coupled with the multimodality of the $f({\bf{x}},{\bf{w}}|{\bf{y}})$ induced by the joint model $f({\bf{x}},{\bf{w}})$. Indeed, adopting a Gibbs sampling strategy to sample iteratively from $f({x}_{n},{w}_{n}|{\bf{y}},{{\bf{x}}}_{\backslash n},{{\bf{w}}}_{\backslash n})$, where **w**_\*n*_ contains all the elements of **w** but ${w}_{n}$, leads to poor mixing properties for the resulting Markov chain and thus prohibitively long chains. Similarly, block Gibbs samplers yield low acceptance rates and also poor mixing properties.

#### Proposed algorithm using variational inference

In this paper, we adopt an approximate Bayesian method and build an approximate distribution $Q({\bf{x}},{\bf{w}})\approx f({\bf{x}},{\bf{w}}|{\bf{y}})$ whose moments are much simpler to evaluate than those of $f({\bf{x}},{\bf{w}}|{\bf{y}})$. In particular, for the identification of the nuclides present in a mixture, one important quantity is ${\text{E}}_{f({\bf{x}},{\bf{w}}|{\bf{y}})}[{\bf{w}}]$, the vector of marginal a posteriori probabilities of presence of each nuclide. For the quantification of the nuclides, interesting quantities are the posterior mean and covariance of **x**, i.e., ${\text{E}}_{f({\bf{x}},{\bf{w}}|{\bf{y}})}({\bf{x}})$ and ${\text{Cov}}_{f({\bf{x}},{\bf{w}}|{\bf{y}})}({\bf{x}})$. While the posterior mean is used as point estimate for the mixing coefficients, the posterior covariance matrix of **x** can be used to assess which sources are the most difficult to quantify. Here, we use the so-called expectation propagation (EP) method^33^ to provide approximate point estimates, e.g., ${\text{E}}_{Q({\bf{x}},{\bf{w}})}({\bf{x}})\approx {\text{E}}_{f({\bf{x}},{\bf{w}}|{\bf{y}})}({\bf{x}})$ and ${\text{E}}_{Q({\bf{x}},{\bf{w}})}[{\bf{w}}]\approx {\text{E}}_{f({\bf{x}},{\bf{w}}|{\bf{y}})}[{\bf{w}}]$, as well as approximations of the covariance of the posterior distribution of **x**, i.e., ${\text{Cov}}_{Q({\bf{x}},{\bf{w}})}({\bf{x}})\approx {\text{Cov}}_{f({\bf{x}},{\bf{w}}|{\bf{y}})}({\bf{x}})$. While less well known than other Variational Bayes (VB) techniques, the EP has several recognized advantages^34^. It is particularly well suited to fast distributed Bayesian inference on partitioned data, giving it a high potential for real-time implementation.

**Figure 8:**
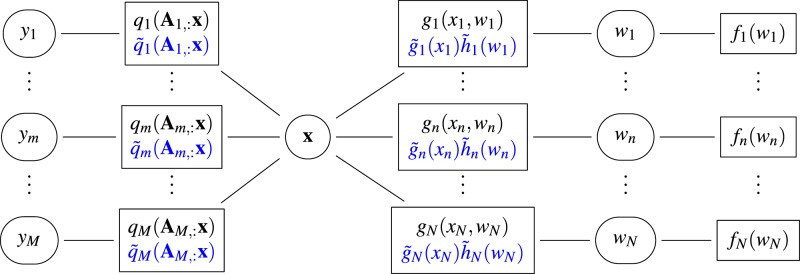
Factor graph used to perform EP-based sparse spectral unmixing. The circles (resp. rectangular boxes) represent the variable (resp. factor) nodes and the approximate factors are shown in blue.

The EP framework used for regression with Gaussian noise^35^ and generalised linear models^36^, approximates each exact factor $f({y}_{m}|{{\bf{A}}}_{m,:}{\bf{x}})={q}_{m}({{\bf{A}}}_{m,:}{\bf{x}})$ (resp. $f({x}_{n}|{w}_{n})={g}_{n}({x}_{n},{w}_{n})$) with a simpler factor ${\tilde{q}}_{m}({{\bf{A}}}_{m,:}{\bf{x}})$ (resp. ${\tilde{g}}_{n}({x}_{n}){\tilde{h}}_{n}({w}_{n})$) so that

5 $$\begin{array}{lll}f({\bf{x}},{\bf{w}}|{\bf{y}}) & \propto  & \mathop{\prod }\limits_{m=1}^{M}\,{q}_{m}({{\bf{A}}}_{m,:}{\bf{x}})\,\mathop{\prod }\limits_{n=1}^{N}\,{g}_{n}({x}_{n},{w}_{n})f({w}_{n})\\  & \approx  & \mathop{\prod }\limits_{m=1}^{M}\,{\tilde{q}}_{m}({{\bf{A}}}_{m,:}{\bf{x}})\,\mathop{\prod }\limits_{n=1}^{N}\,{\tilde{g}}_{n}({x}_{n}){\tilde{h}}_{n}({w}_{n})f({w}_{n})=Q({\bf{x}},{\bf{w}}),\end{array}$$

where all the approximate factors belong to the same family of distributions. Here, in a similar fashion to the work by Hernandez-Lobato *et al*.^37^, the approximate factors dependent on **x** are Gaussian and those associated with each ${w}_{n}$ are discrete probabilities (see Fig. 8). This choice allows a more computationally attractive EP algorithm and direct access to the moments of the posterior distribution. Moreover, it is important to note that using the splitting $f({x}_{n}|{w}_{n})\approx {\tilde{g}}_{n}({x}_{n}){\tilde{h}}_{n}({w}_{n})$, the approximate distribution $Q({\bf{x}},{\bf{w}})$ can be written $Q({\bf{x}},{\bf{w}})={Q}_{x}({\bf{x}}){Q}_{w}({\bf{w}})$, i.e., the approximation does not explicitly capture the correlation a posteriori between **x** and **w**. Nonetheless, this type of separable approximation is classically used in variational inference and the parameters of ${Q}_{x}(\cdot )$ and ${Q}_{w}(\cdot )$ are in practice highly dependent. To optimize $Q({\bf{x}},{\bf{w}})$ so that $f({\bf{x}},{\bf{w}}|{\bf{y}})\approx Q({\bf{x}},{\bf{w}})$, EP sequentially refines the factors ${\{{\tilde{q}}_{m}({{\bf{A}}}_{m,:}{\bf{x}})\}}_{m}$ and ${\{{\tilde{g}}_{n}({x}_{n}),{\tilde{h}}_{n}({w}_{n})\}}_{n}$ by minimizing the following Kullback-Leibler (KL) divergences

6 $$\{\begin{array}{ll}\mathop{\text{min}}\limits_{{\tilde{q}}_{m}}KL({q}_{m}({{\bf{A}}}_{m,:}{\bf{x}}){Q}^{\backslash m}({\bf{x}},{\bf{w}})\parallel {\tilde{q}}_{m}({{\bf{A}}}_{m,:}{\bf{x}}){Q}^{\backslash m}({\bf{x}},{\bf{w}})), & \forall m=1,\ldots ,M\\ \mathop{\text{min}}\limits_{{\tilde{g}}_{n},{\tilde{h}}_{n}}KL({g}_{n}({x}_{n},{w}_{n}){Q}^{\backslash n}({\bf{x}},{\bf{w}})\parallel {\tilde{g}}_{n}({x}_{n}){\tilde{h}}_{n}({w}_{n}){Q}^{\backslash n}({\bf{x}},{\bf{w}})), & \forall n=1,\ldots ,N\end{array}$$

where the so-called cavity distributions satisfy $${Q}^{\backslash m}({\bf{x}},{\bf{w}})=Q({\bf{x}},{\bf{w}})/{\tilde{q}}_{m}({{\bf{A}}}_{m,:}{\bf{x}})$$ and $${Q}^{\backslash n}({\bf{x}},{\bf{w}})=Q({\bf{x}},{\bf{w}})/$$$$({\tilde{g}}_{n}({x}_{n}){\tilde{h}}_{n}({w}_{n}))$$. Solving the first row of Eq. (6) reduces to matching the mean and covariance of $${Q}_{x}({\bf{x}})$$ and of the so-called tilted distributions $$\int \,{q}_{m}({{\bf{A}}}_{m,:}{\bf{x}}){Q}^{\backslash m}({\bf{x}},{\bf{w}})d{\bf{w}},\forall m$$. In the work by Ko *et al*.^38^, the authors showed that these problems can be solved analytically by computing sequentially one-dimensional integrals (see also^11^ for additional details). The second row of Eq. (6) can be solved by using the method presented by Hernández-Lobato and colleagues^37^. Since the approximation of $${g}_{n}({x}_{n},{w}_{n})$$ is separable ($${\tilde{g}}_{n}({x}_{n}){\tilde{h}}_{n}({w}_{n})$$), it is sufficient to compute the mean of $${w}_{n}$$ with respect to the tilted distribution $$\int \,{g}_{n}({x}_{n},{w}_{n}){Q}^{\backslash n}({\bf{x}},{\bf{w}})d{\bf{w}}$$ as well as the mean and covariance of the tilted distribution $$\int \,{g}_{n}({x}_{n},{w}_{n}){Q}^{\backslash n}({\bf{x}},{\bf{w}})d{\bf{w}}$$, which in turn reduces to computing the first and second-order moments of $$\int \int \,{g}_{n}({x}_{n},{w}_{n}){Q}^{\backslash n}({\bf{x}},{\bf{w}})d{\bf{w}}d{{\bf{x}}}_{\backslash n}$$, with respect to $${x}_{n}$$. This last distribution can be shown to be a Bernoulli truncated Gaussian distribution whose moments can be computed analytically. Finally, in a similar fashion to the procedure proposed by Hernández-Lobato and colleagues^37^, we used a damping strategy to reduce convergence issues. We fixed the damping factor to $$\varepsilon =0.7$$ and did not observe convergence issues with this value. When the algorithm has converged, we obtain $${Q}_{x}({\bf{x}})$$ which is a multivariate Gaussian distribution, and $${Q}_{w}({\bf{w}})$$ which is a product of $$N$$ independent Bernoulli distributions, whose parameters have been optimized via the EP algorithm such that $$f({\bf{x}},{\bf{w}}|{\bf{y}})\approx Q({\bf{x}},{\bf{w}})$$ The approximate posterior mean and covariance matrix of $$x$$ are given by the mean and covariance matrix of $${Q}_{x}({\bf{x}})$$, respectively. To compute the estimated mixture fractions in Eq. (1), from any estimated mixture coefficients $$\hat{{\bf{x}}}$$ (e.g., by MMSE_*BTG*_, MAP_*L*1_ or MMSE_*L*1_), we then consider $$\hat{{\bf{z}}}=\hat{{\bf{x}}}/\parallel \hat{{\bf{x}}}{\parallel }_{1}$$. The parameters of the Bernoulli distributions in $${Q}_{w}({\bf{w}})$$ provide the approximate marginal posterior probabilities of presence, for each source. Thus, the source identification can be preformed using $${Q}_{w}({\bf{w}})$$, without resorting to thresholding the estimated mixture coefficients. Choosing the most appropriate decision rule for the source identification based on the marginal posterior distribution ultimately reduces to choosing an acceptable threshold for the probability of presence. Here, we consider a detection when the probability of presence is larger than the probability of absence, effectively using a marginal MAP criterion. If costs associated with the probabilities of false alarm and misdetection are available for each source, similar decision rules can also be easily derived using the output of the proposed method, based on a minimum cost criterion instead of the marginal MAP criterion. However, the study of such decision rules is out of scope of this paper. The current version of the algorithm is available at the url: https://gitlab.com/yaltmann/sparse_unmixing_poisson_noise_ep.
